# Supplementary material for: Immunodeficiencies Push Readmissions in Malignant Tumor Patients: A Retrospective Cohort Study Based on the Nationwide Readmission Database
Source: Cancers (Basel). 2022 Dec 23;15(1):88. doi: 10.3390/cancers15010088 (PMC9817498; doi:10.3390/cancers15010088)
Supplement: Supplementary file 1 [file cancers-15-00088-s001.zip › cancers-2078696-supplementary.pdf]

Supplemental Table S1. The International Classification of Diseases, Tenth Revision, Clinical Modification (ICD-10-CM) Codes used in our study.

	Diseases	Codes
**Primary Immunodeficiency**	Immunodeficiencies affecting cellular and humoral immunity	D810, D811, D812, D8131, D813, D8130, D8132, D8139, D816, D817, D819, D8189
	Combined immunodeficiencies with associated or syndromic features	D814, D815, D820, D821, D822, D824, D828, D829, D8481, D84821, D84822, D512
	Predominantly antibody deficiencies	D800, D801, D802, D803, D804, D805, D806, D807, D808, D809, D830, D831, D832, D838, D839
	Diseases of immune dysregulation	D823, D840, E70330,
	Complement deficiencies	D841
	Other immunodeficiencies	D848, D8489, D849
**Secondary Immunodeficiency**	Acquired immune deficiency syndrome	B20, B9735, O98711, O98712, O98713, O98719, O9872, O9873
**Malignancy**	Bladder	C670- C679
	Brain & other nervous	C700, C701, C709-C721, C7220-C7222, C7230-C7232, C7240-C7242, C7250, C7259, C729-
	Breast	C50011, C50012, C50019, C50021, C50022, C50029, C50111, C50112, C50119, C50121, C50122, C50129, C50211, C50212, C50219, C50221, C50222, C50229, C50311, C50312, C50319, C50321, C50322, C50329, C50411, C50412, C50419, C50421, C50422, C50429, C50511, C50512, C50519, C50521, C50522, C50529, C50611, C50612, C50619, C50621, C50622, C50629, C50811, C50812, C50819, C50821, C50822, C50829, C50911, C50912, C50919, C50921, C50922, C50929
	Cervix	C530, C531, C538, C539
	Colorectal	C180, C182- C189, C19, C20
	Esophagus	C153- C155, C158, C159
	Leukemia	C9100-C9102, C9110-C9112, C9130-C9132, C9140-C9142, C9150-C9152, C9160-C9162, C9190-C9192, C91A0-C91A2, C91Z0-C91Z2, C9200-C9202, C9210-C9212, C9220-C9222, C9240-C9242, C9250-C9252, C9260-C9262, C9290-C9292, C92A0-C92A2, C92Z0-C92Z2, C9300-C9302, C9310-C9312, C9330-C9332, C9390-C9392, C93Z0-C93Z2, C9400-C9402, C9420-C9422, C9430-C9432, C9440-C9442, C946, C9480-C9482, C9500- C9502, C9510-C9512, C9590-C9592
	Liver	C220-C224, C227, C228
	Lung	C3400-C3402, C3410-C3412, C342, C3430-C3432, C3480-C3482, C3490-C3492
	non-Hodgkin lymphoma	C8200-C8269, C8280-C8299, C8300-C8319, C8330- C8339, C8350-C8359, C8370- C8399, C8400-C8419, C8440-C8449, C8460-C8479, C8490-C8499, C84A0-C84A, C84Z0-C84Z9, C8510- C8529, C8580-C8599, C860–C866, C880, C882-C884, C888, C889
	Ovary	C561, C562, C569
	Pancreas	C250-C254, C257- C259

	Prostate	C61
	Stomach	C160-C166, C168, C169
	Thyroid	C73
**Readmitted causes**	Anemia	D500, D501, D508-D513, D518-D521, D528-D532, D538, D539, D550-D553, D558-D565, D568, D569, D5700-D5703, D5709, D571, D5720, D57211-D57213, D57218, D57219, D573, D5740, D57411-D57413, D57418, D57419, D5742, D57431-D57433, D57438, D57439, D5744, D57451-D57453, D57458, D57459, D5780, D57811, D57812, D57813, D57818, D57819, D580, D581, D582, D588, D589, D590, D591, D5910, D5911-D5913, D5919, D592-D596, D598-D601, D608, D609, D6101, D6109, D611-D613, D61810, D61811, D61818, D6182, D6189, D619, D62, D630, D631, D638, D640-D644, D6481, D6489, D649,
	Chronic obstructive pulmonary disease, COPD	J410, J411, J418, J42, J430-J432, J438-J441, J449
	Fluid and electrolyte disorders	E860, E861, E869-E876, E8770, E8771, E8779, E878
	Gastro-esophageal reflux disease	K210, K2100, K2101, K219
	Heart diseases	I200, I201, I208, I209, I2101, I2102, I2109, I2111, I2119, I2121, I2129, I213, I214, I219, I21A1, I21A9, I220-I222, I228, I229, I230-I241, I248, I249, I2510, I25110, I25111, I25118, I25119, I252, I253, I2541, I2542, I255, I256, I25700, I25701, I25708, I25709, I25710, I25711, I25718-I25721, I25728-I25731, I25738, I25739, I25750, I25751, I25758-I25761, I25768, I25769, I25790, I25791, I25798, I25799, I25810-I25812, I2582, I2583, I2584, I2589, I259
	Infection	A000-A499, A510-A99
	Kidney failure	N170-N172, N178, N179, N184-N186, N19
	Pneumonia	J120-J123, J1281, J1282, J1289, J129, J13, J14, J150, J151, J1520, J15211, J15212, J1529, J153, J154, J155-J160, J168, J17, J180-J182, J188, J189
	Secondary malignancies	C770, C771, C772, C773, C774, C775, C778, C779, C7800, C7801, C7802, C781, C782, C7830, C7839, C784-C787, C7880, C7889, C7900, C7901, C7902, C7910, C7911, C7919, C792, C7931, C7932, C7940, C7949, C7951, C7952, C7960, C7961, C7962, C7970, C7971, C7972, C7981, C7982, C7989, C799
